# Seasonal Dynamics of Photochemical Performance of PS II of Terrestrial Mosses from Different Elevations

**DOI:** 10.3390/plants10122613

**Published:** 2021-11-28

**Authors:** Jiewei Hao, Xueyan Xu, Lina Zhang

**Affiliations:** 1 School of Life Sciences, The Chinese University of Hong Kong, Shatin, Hong Kong, China; 2 Center for Eco-Environmental Restoration Engineering of Hainan Province, Hainan University, 58 Renmin Road, Haikou 570228, China; xuxueyan@hvust.edu.cn; 3 Center for Terrestrial Biodiversity of the South China Sea, Hainan University, 58 Renmin Road, Haikou 570228, China

**Keywords:** chlorophyll fluorescence, ecophysiology, environmental stress, photosystem II, terrestrial mosses

## Abstract

Mosses are critical components of tropical forest ecosystems and have multiple essential ecological functions. The drying and rehydrating and often hot environments in tropical regions present some of the greatest challenges for their photosynthetic activities. There is limited knowledge available on the physiological responses to the changing environments such as temperature and water pattern changes for terrestrial mosses. We examined the seasonal dynamics of photochemical performance of PS II through the measuring of chlorophyll fluorescence of 12 terrestrial mosses in situ from five different elevations by Photosynthesis Yield Analyzer MINI-PAM-II, along with the seasonal changes of climatic factors (air temperature, dew point, relative humidity and rainfall), which were collected by local weather stations and self-deployed mini weather stations. The results showed a great seasonality during observing periods, which, mainly the changes of rainfall and relative humidity pattern, presented significant impacts on the photochemical performance of PS II of terrestrial mosses. All these tested moss species developed a suitable regulated and non-regulated strategy to avoid the detrimental effect of abiotic stresses. We found that only *Hypnum plumaeforme*, *Pterobryopsis crassicaulis* and *Pogonatum inflexum* were well adapted to the changes of habitat temperature and water patterns, even though they still experienced a lower CO_2_ assimilation efficiency in the drier months. The other nine species were susceptible to seasonality, especially during the months of lower rainfall and relative humidity when moss species were under physiologically reduced PS II efficiency. *Anomobryum julaceum*, *Pogonatum neesii*, *Sematophyllum subhumile*, *Pseudotaxiphyllum pohliaecarpum* and *Leucobryum boninense*, and especially *Brachythecium buchananii*, were sensitive to the changes of water patterns, which enable them as ideal ecological indicators of photosynthetic acclimation to stressed environments as a result of climate change.

## 1. Introduction

Photosynthesis is particularly sensitive to adverse environmental factors, such as high air temperature and vapor pressure deficits [1], making photosynthetic measurements an important component in environmental and ecological studies. To avoid the abiotic stresses, land plants have developed photoprotective mechanisms that enable them to dissipate excess excitation energy as heat via the so-called non-photochemical quenching (NPQ) mechanism [2,3,4]. Even though bryophytes (liverworts, hornworts and mosses) are the earliest diverging lineages of the extant land plants [5,6,7], they still face great challenges of the cycle of drying and rehydrating and often high temperatures in tropical regions. Given the poikilohydric nature of their water relations, bryophyte carbon dynamics are affected by the respiratory demands of desiccation and rehydration as well as by the photosynthetic assimilation of active thallus when hydrated [8]. Temperature, one of the key factors determining metabolism, growth, and distribution in plants, exerts asymmetrical effects on respiration and photosynthesis [9].

When photosynthetic organisms are in a normal physiological state, ~5% of harvested light energy by Photosystem II (PSII) is reemitted as chlorophyll fluorescence [10]. Changes in chlorophyll fluorescence induced by illumination of dark-acclimated leaves can be qualitatively correlated with changes in CO_2_ assimilation. Fluorescence emissions in photosynthetic organisms can be correlated to the photosynthetic rates [11,12]. Consequently, the interest in identifying feedbacks of plants to habitat stresses by measuring chlorophyll fluorescence is increasing. Pulse-amplitude-modulated (PAM) fluorometry is one of the most common techniques used to study the induction and quenching of chlorophyll fluorescence in physiological studies [13]. PAM fluorescence system is a powerful and widely used tool in the study of plant photosynthesis under abiotic stresses [12,14,15,16,17,18,19]. Effective quantum yield of PS II (ΔF/Fm′) derived from chlorophyll fluorescence measurement is a sensitive indicator of plant photosynthetic performance. Its decline is regarded as a reduction in PS II efficiency. Seasonal dynamics in response to an effective quantum yield of PS II photochemistry (Y(II)) to photosynthetically active radiation (PAR) have been deduced to reflect changes in the physiological status of plants in changing environments. The component of the Stern–Volmer type NPQ that plays a crucial role under fluctuating light conditions is the fast component (ΔpH-PsbS dependent-qE3 or zeaxanthin dependent-qZ4) [20,21], which increased with increasing light intensities in all the seasons. In addition, it is a simple, rapid and non-destructive widely utilized technique [11,22,23,24]; meanwhile, it can be performed on tiny plants such as mosses [25].

Mosses are critical components of tropical forest ecosystems [26] and have essential ecological functions [27]. The drying and rehydrating and hot environments in tropical regions present some of the greatest challenges for bryophyte photosynthetic activities [8]. Meanwhile, as climate conditions in the tropics are expected to become hotter and drier [28,29], many moss species might be negatively affected or even at risk of extinction. Therefore, the ecophysiological responses of these plants to seasonal changes of temperature and water patterns should be explored, particularly under global climate change. Here in southern China, we firstly investigated the seasonal dynamics of the potential photochemical performance of PS II of terrestrial mosses in their current habitat to assess their acclimation to the seasonal fluctuations of temperature and water patterns during the growing season.

## 2. Results

### 2.1. Seasonal Changes of Environmental Factors

**Figure 1:**
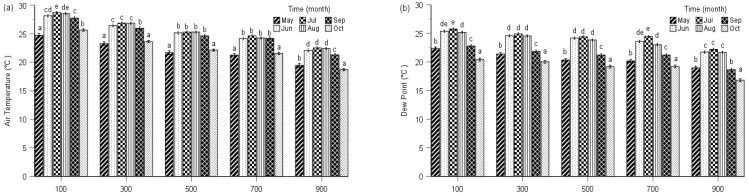

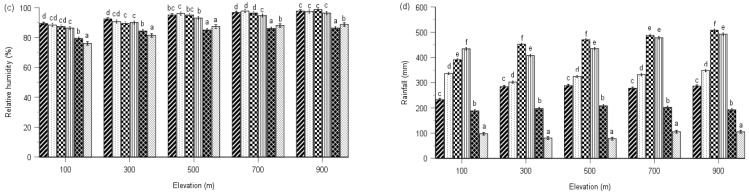
Seasonal changes of mean air temperature (**a**), dew point (**b**), relative humidity (**c**) and rainfall (**d**) at five elevations during the 2019 growing season. Bars of same elevation marked by different letters are significantly different (*p* < 0.05). Error bars indicate the 95% confidence interval (*n* = 3).

To monitor the photosynthetic performance of terrestrial moss species at five different elevations on Tai Mo Shan, we recorded several PSII parameters during the growing season in the year 2019 along with changes in monthly average air temperature, dew point, relative humidity and rainfall of the five study elevations (Figure 1). 

Multi-way ANOVAs showed significant effects of observing time on air temperature, dew point, relative humidity and rainfall for all five elevations (Table A1). The dynamics of air temperature, dew point, relative humidity and rainfall exhibited similar seasonal patterns among the five elevations (Figure 1). For simplicity, here we only present the detailed climatic factors at 900 m. 

At 900 m, the mean air temperature of July (22.5 °C), August (22.4 °C) and June (22.1 °C) was significantly higher than that of September (21.3 °C), May (19.5 °C) and October (18.7 °C), with significant differences between that of September, May and October. The mean dew point showed a similar trend compared with air temperature, with mean values of July (22.0 °C), June (21.7 °C) and August (21.6 °C) significantly higher than that of May (19.0 °C), September (18.8 °C) and October (17.0 °C). We detected the relative humidity of July (98.3%), May (97.7%), June (97.3%) and August (96.3%) was significantly higher than that of October (88.7%) and September (86.3%). The mean rainfall of July (507 mm) was significantly higher than August (492 mm), June (348 mm), May (286 mm), September (192 mm) and October (105 mm), and significant differences were measured between the other observing times (Figure 1).

### 2.2. PSII Photochemistry

**Figure 2:**
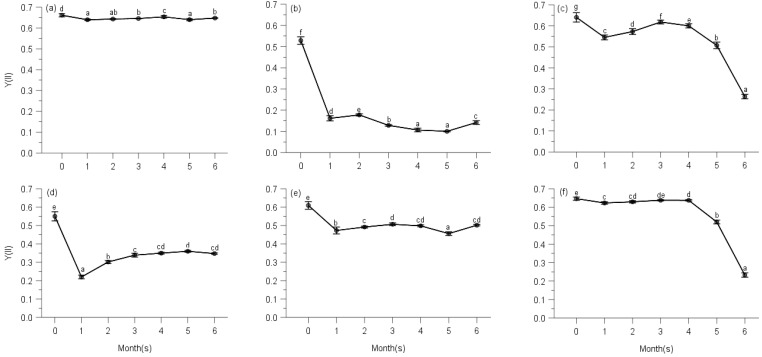
Changes of photochemical quantum yield of PS II (Y(II)) of Hypnum plumaeforme (**a**), Brachythecium buchananii (**b**), Thuidium glaucinoides (**c**), Anomobryum julaceum (**d**), Pterobryopsis crassicaulis (**e**) and Pseudosymblepharis angustata (**f**) from 900 m during 6 months of measurements. 0: the initial photochemical quantum yield of PS II, i.e., maximum photochemical quantum yield Fv/Fm defined as control. Numbers 1 to 6 mean from May to October, which is employed by following Figure 3, Figure 4, Figure 5, Figure 6, Figure 7, Figure 8, Figure 9, Figure 10 and Figure 11. Values marked by different letters are significantly different (*p* < 0.05). Error bars indicate 95% confidence interval (*n* = 3).

**Figure 3:**
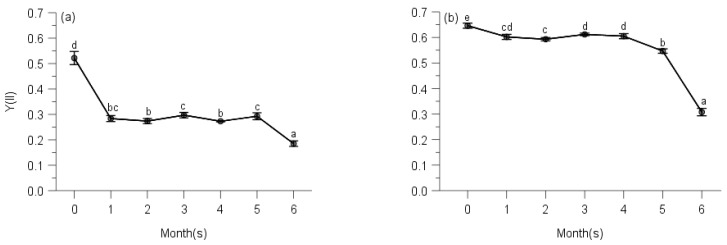
Changes of photochemical quantum yield of PS II of *Pogonatum neesii* (**a**) and *Leucobryum scabrum* (**b**) from 700 m during 6 months of measurements. 0: the initial photochemical quantum yield of PS II, i.e., maximum photochemical quantum yield Fv/Fm defined as control. Values marked by different letters are significantly different (*p* < 0.05). Error bars indicate 95% confidence interval (*n* = 3).

**Figure 4:**
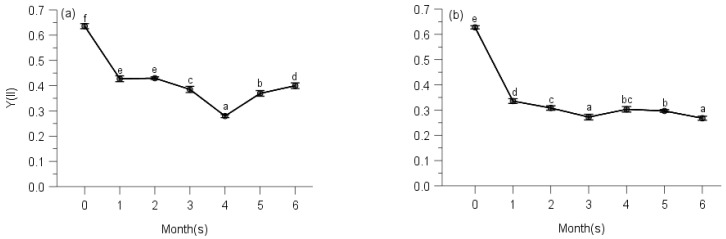
Changes of photochemical quantum yield of PS II of *Sematophyllum subhumile* (**a**) and *Leucobryum boninense* (**b**) from 500 m during 6 months of measurements. 0: the initial photochemical quantum yield of PS II, i.e., maximum photochemical quantum yield Fv/Fm defined as control. Values marked by different letters are significantly different (*p* < 0.05). Error bars indicate 95% confidence interval (*n* = 3).

**Figure 5:**
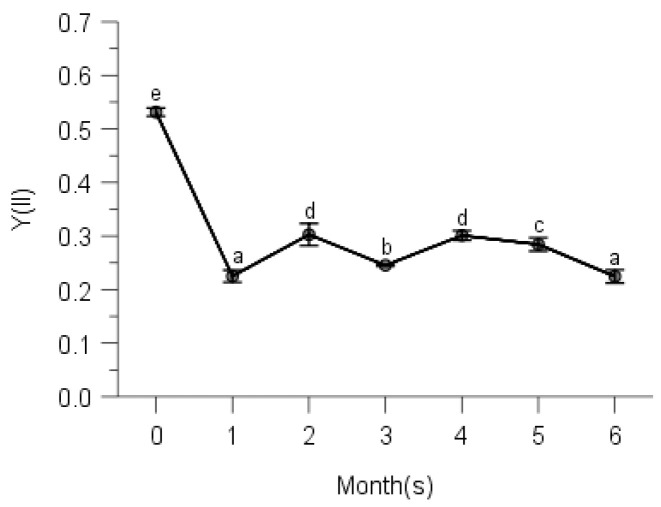
Changes of photochemical quantum yield of PS II of *Pseudotaxiphyllum pohliaecarpum* from 300 m during 6 months of measurements. 0: the initial photochemical quantum yield of PS II, i.e., maximum photochemical quantum yield Fv/Fm defined as control. Values marked by different letters are significantly different (*p* < 0.05). Error bars indicate 95% confidence interval (*n* = 3).

**Figure 6:**
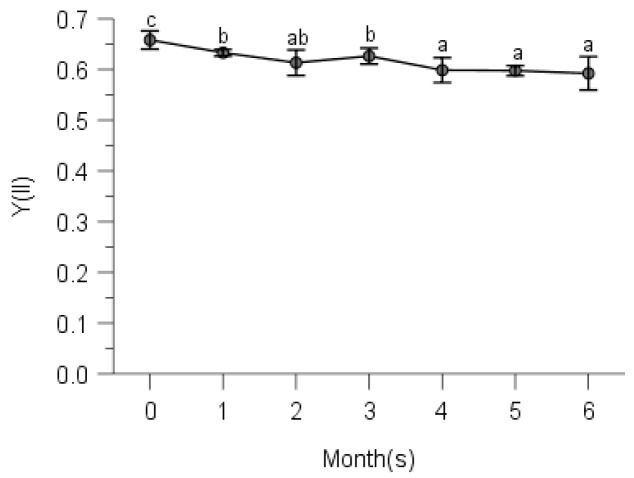
Changes of photochemical quantum yield of PS II of *Pogonatum inflexum* from 100 m during 6 months of measurements. 0: the initial photochemical quantum yield of PS II, i.e., maximum photochemical quantum yield Fv/Fm defined as control. Values marked by different letters are significantly different (*p* < 0.05). Error bars indicate 95% confidence interval (*n* = 3).

Multi-way ANOVAs showed significant effects of observing time on effective quantum yield of PS II (Y(II)) for all species at five elevations (Table A2). Maximal quantum yields of PS II (Fv/Fm defined as control) were significantly higher than effective quantum yields at any other measuring time for all moss species (Table A2, Figure 2, Figure 3, Figure 4, Figure 5 and Figure 6).

From Figure 2, we know that *Hypnum plumaeforme*, *Brachythecium buchananii*, *Thuidium glaucinoides*, *Anomobryum julaceum* and *Pterobryopsis crassicaulis* from 900 m responded to the environmental factors in three different general ways. Y(II) of *H*. *Plumaeforme* in August (98.8% of control) was significantly better than those of October, July, June, September and May; no significant differences existed between June, July and October, so as those between September, June and May. The lowest Y(II) in May, however, still reached 96.7% of control (Figure 2a). *B*. *buchananii* was especially susceptible to the abiotic factors. The highest Y(II) of it only reached 33.6% of control in June, which was statistically higher than those of May, October, July, August and September, and significant differences were measured between these five months, except August (lowest, 20.1% of control) and September (lowest, 18.9% of control) (Figure 2b). The highest Y(II) of *T*. *glaucinoides* was in July (96.5% of control), statistically higher than those of August, June, May, September and October (lowest, 41.1%); and significant differences were found between the other five observing months (Figure 2c). Y(II) of *A*. *julaceum* in September (65.4% of control) was significantly greater than those of July, June and May (lowest, 40% of control); no significant differences existed between September, August and October, compared to that of August, October and July (Figure 2d). We detected the highest Y(II) of *P*. *crassicaulis* in July (83.2% of control), which was significantly greater than those of June, May and September (lowest, 74.8% of control); however, Y(II) of October with lower rainfall and relative humidity was not significantly lower than those of July, August and June (Figure 2e). The highest Y(II) of *P*. *angustata* was found in July (98.7% of control), which was statistically better than those of May, September and October (lowest, 36.0% of control). We measured no significant differences between July, August and June, as that between June and May (Figure 2f).

Even though the *Pogonatum neesii* and *Leucobryum scabrum* were from the same habitat at 700 m, they responded to the seasonal changes of climate totally differently. The highest Y(II) of *P*. *neesii* was in July (56.9% of control), which was significantly higher than that of June, August and October (lowest, 35.5% of control). No significant differences were detected between the wetter months of July and May and the relatively drier month of September (Figure 3a). *L*. *scabrum* obtained the best Y(II) in July (94.7% of control), which was significantly higher than those of June, September and October (lowest, 47.6% of control). In the drier months, it achieved the least Y(II) (Figure 3b).

We observed the highest Y(II) of *Sematophyllum subhumile* in June (67.6% of control), which was significantly better than those of October, July, September and August (lowest, 44.0% of control). The Y(II) of October under the lowest rainfall and relative humidity was significantly higher than those of July and August with better environmental conditions. No significant differences existed between that of June and May (Figure 4a), except the other 4 months. *Leucobryum boninense* achieved the best Y(II) in May (53.5% of control), significantly higher than those of June, August, September, July and October (lowest, 42.6% of control) (Figure 4b).

The highest Y(II) of *Pseudotaxiphyllum pohliaecarpum* was detected in June (only 57.0% of control), which was significantly greater than those of September, July, May and October (lowest, 42.3% of control); however, under the higher rainfall and relative humidity of July, the Y(II) was relatively lower (Figure 5).

Y(II) of *Pogonatum inflexum* in May (96.2% of control) was significantly better than those of August, September and October (lowest, 90.0% of control); even under the lower rainfall and drier months of September and October, it still achieved better performance such as that of June and August (Figure 6).

### 2.3. Regulated and Non-Regulated Energy Dissipation

**Figure 7:**
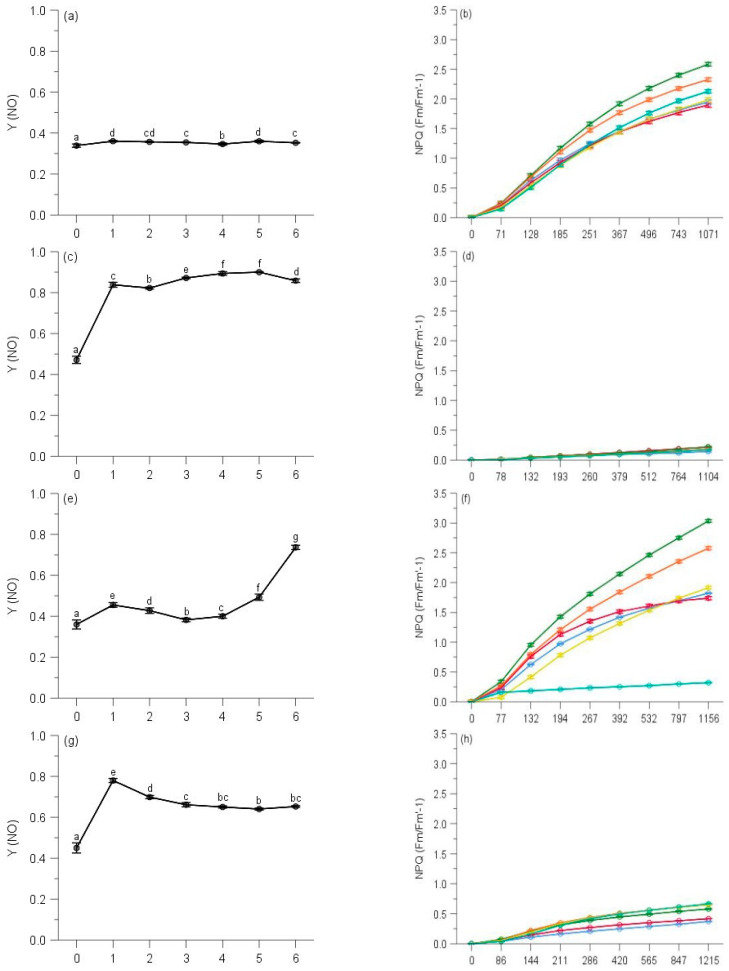

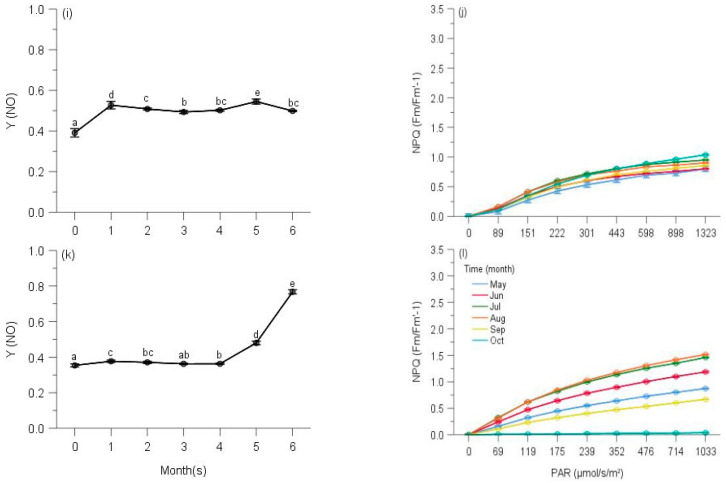
Seasonal changes of Y(NO) (**a**) and NPQ (**b**) of *Hypnum plumaeforme*; Y(NO) (**c**) and NPQ (**d**) of *Brachythecium buchananii*; Y(NO) (**e**) and NPQ (f) of *Thuidium glaucinoides*; Y(NO) (**g**) and NPQ (**h**) of *Anomobryum julaceum*; Y(NO) (**i**) and NPQ (**j**) of *Pterobryopsis crassicaulis*; Y(NO) (**k**) and NPQ (**l**) of *Pseudosymblepharis angustata* from 900 m during 6 observing months. 0: the initial quantum yield of non-regulated heat dissipation of PS II (defined as control). Values marked by different letters are significantly different (*p* < 0.05). Error bars indicate 95% confidence interval (*n* = 3).

**Figure 8:**
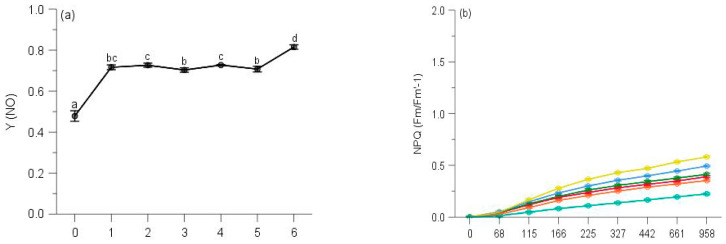

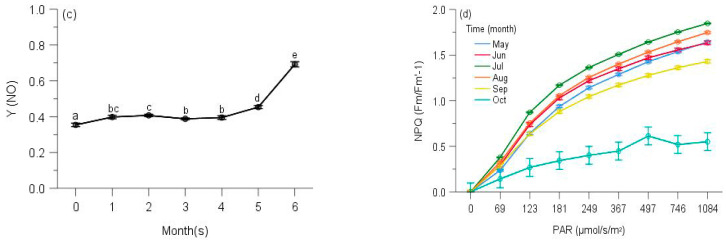
Seasonal changes of Y(NO) (**a**) and NPQ (**b**) of *Pogonatum neesii* and Y(NO) (**c**) and NPQ (**d**) of *Leucobryum scabrum* from 700 m during 6 observing months. 0: the initial quantum yield of non-regulated heat dissipation of PS II (defined as control). Values marked by different letters are significantly different (*p* < 0.05). Error bars indicate 95% confidence interval (*n* = 3).

**Figure 9:**
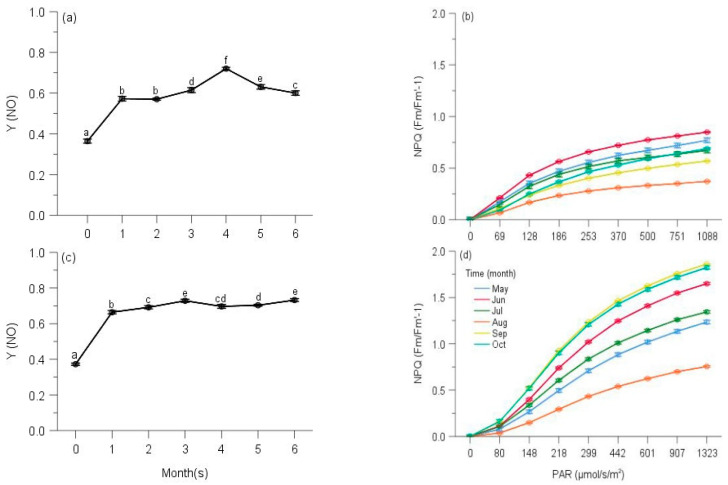
Seasonal changes of Y(NO) (**a**) and NPQ (**b**) of *Sematophyllum subhumile*, and Y(NO) (**c**) and NPQ (**d**) of *Leucobryum boninense* from 500 m during 6 observing months. 0: the initial quantum yield of non-regulated heat dissipation of PS II (defined as control). Values marked by different letters are significantly different (*p* < 0.05). Error bars indicate 95% confidence interval (*n* = 3).

**Figure 10:**
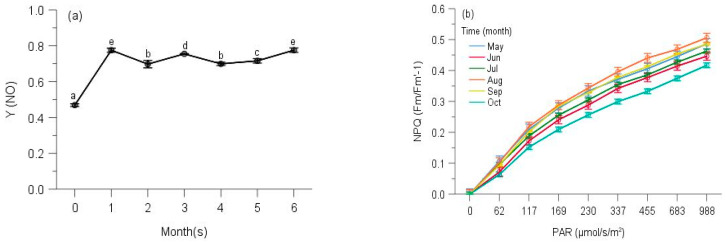
Seasonal changes of Y(NO) (**a**) and NPQ (**b**) of *Pseudotaxiphyllum pohliaecarpum* from 300 m during 6 observing months. 0: the initial quantum yield of non-regulated heat dissipation of PS II (defined as control). Values marked by different letters are significantly different (*p* < 0.05). Error bars indicate 95% confidence interval (*n* = 3).

**Figure 11:**
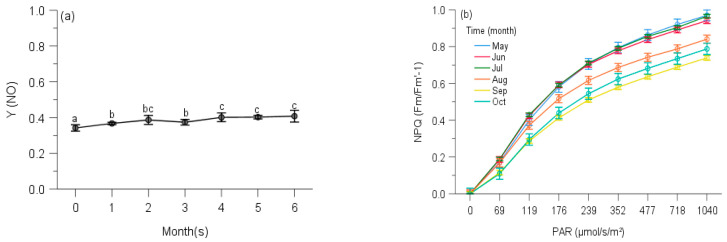
Seasonal changes of Y(NO) (**a**) and NPQ (**b**) of *Pogonatum inflexum* from 100 m during 6 observing months. 0: the initial quantum yield of non-regulated heat dissipation of PS II (defined as control). Values marked by different letters are significantly different (*p* < 0.05). Error bars indicate 95% confidence interval (*n* = 3).

Multi-way ANOVAs showed significant effects of sampling time on Y(NO) (non-regulated losses of excitation energy including heat dissipation and fluorescence emission) and Stern–Volmer type NPQ (parameters of non-photochemical quenching) of PS II for all species at five elevations (Table A3). Changes in Y(NO) of each species showed a reverse trend compared with counterpart ΔF/Fm′ of moss species, and the initial Y(NO) was significantly lower than that of any other month for all moss species (Table A3, Figure 7, Figure 8, Figure 9, Figure 10 and Figure 11). 

Y(NO) of *H. plumaeforme* in May was significantly higher than those of July, October and August (Figure 7a). In all six observing months, the fast component did not reach a static phase even at 1017 μmol/s/m^2^. The Stern–Volmer type NPQ in July was higher than those of August, October, May, September and June, the latter two of which were significantly smaller and overall slower than those of other months (Figure 7b). Y(NO) of *B. buchananii* in September was remarkedly greater than those of July, October, May and June, and significant differences were measured between July, October, May and June, except that between August and September (Figure 7c). We did not observe the stationary phase during all six months, even though all the NPQ values were lower than 0.25. The NPQs in June and July were higher than those of August, September, October and May, the latter two of which were significantly smaller and overall slower than those of other months (Figure 7d). Y(NO) of *T. glaucinoides* in October was significantly greater than those of September, May, June, August and July, and significant differences were observed between the other five months (Figure 7e). In October, the fast component obtained a stationary phase when actinic light was at 77 μmol/s/m^2^. The highest NPQ was in July, which was significantly higher than those of August, June, May, September and October, which was significantly smaller and overall slower due to the smaller amplitude of the fast component (12.9% of August) (Figure 7f). 

Y(NO) of *A. julaceum* in May was significantly better than those of June, July, October, August and September, and significant differences were not found between July, October, August, similar to those of October, August and September (Figure 7g). In all six observing months, we found no stationary phase. The NPQ in August was higher than that of September, October, July, June and May (51.8% of August), which was significantly smaller and overall slower than that of other months (Figure 7h). We detected the highest Y(NO) of *P. crassicaulis* in September, which was significantly greater than those of May, June, August, October and July, and no significant differences were detected between June, August and October, similar to those of August, October and July (Figure 7i). In all six observing months, the fast component did not reach a static phase even at 1323 μmol/s/m^2^. The NPQ in July was greater than those of October, August, September, June and May (77.0% of July), which was significantly smaller and overall slower (Figure 7j). The highest Y(NO) of *P. angustata* was found in October, which was significantly bigger than those of September, May, June, August and July (Figure 7k). In October, a stationary phase gained at 69 μmol/s/m^2^. The highest NPQ was in August, which was significantly greater than those of July, June, May, September and October. The latter five were significantly smaller and overall slower due to the smaller amplitude of the fast component (only 2.1% of August) (Figure 7l).

Similar to the responses of their corresponding Y(II), the two species showed the different trends of Y(NO). Y(NO) of *P. neesii* in October was significantly higher than those of August, June, May, September and July (Figure 8a). In all observing months, no stationary phase was observed at 958 μmol/s/m^2^. The highest NPQ was in September, which was significantly higher than those of May, July, June, August and October, which was notably smaller and overall slower than the other observing months (Figure 8b). The Y(NO) of *L. scabrum* in October was significantly higher than those of September, June, May, August and July (Figure 8c). A stationary phase achieved 1084 μmol/s/m^2^ in October. The NPQ in July reached the highest component than those of August, June, May, September and October, which was remarkably smaller and overall slower due to the smaller amplitude of the fast component (31.2% of July) (Figure 8d).

We observed the highest Y(NO) of *S. subhumile* in August, which was significantly greater than those of September, July, October, May and June, and significant differences were not measured between May and June (Figure 9a), except those of the other 4 months. In August, a relatively stationary phase was obtained when measuring light was 1088 μmol/s/m^2^. The NPQ in June reached the highest component compared to. May, July, October, September and August (Figure 9b). Y(NO) of *L. boninense* in October was significantly bigger than those of September, August, June and May, and no significant differences were detected between those of October and July, September and August, similarly to that of August and June (Figure 9c). In all observing months except August, no stationary phase was observed even when actinic light was 1323 μmol/s/m^2^. The NPQ in September obtained the highest component compared to October, June, July, May and August, which was markedly smaller and overall slower than those of the other months observed (Figure 9d).

The highest Y(NO) of *P. pohliaecarpum* was detected in October, which was significantly better than those of May, July, September, August and June (Figure 10a). In all observing months, the stationary phase was not achieved even at 988 μmol/s/m^2^. The NPQ in August yielded a greater component than those of May, September, July, June and October, which was notably smaller and overall slower than those of the other months observed (Figure 10b).

The Y(NO) of *P. inflexum* in October was significantly higher than the other five months (Figure 11a). In all observing months, no stationary phase was observed even at 1040 μmol/s/m^2^. The NPQ in July, May and June gained a higher component than those of August, October and September (Figure 11b), but inducible NPQ in September was remarkably smaller and overall slower than the other months observed.

## 3. Discussion

**Figure 12:**
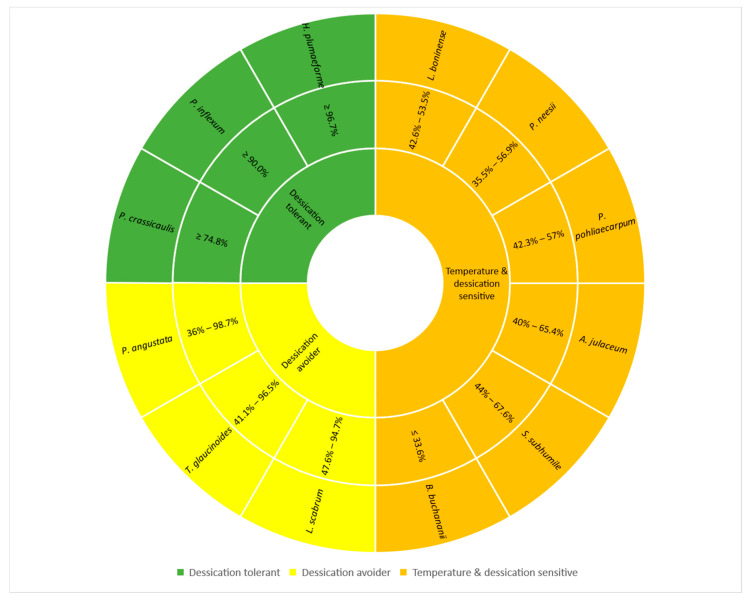
Twelve species grouped into 3 types of responses to seasonal fluctuations of climatic factors based on their photochemical efficiency of PS II: desiccation tolerant, desiccation avoider and temperature and desiccation sensitive.

The results of multi-way ANOVAs showed a strong seasonality during observing periods with significant differences. A hump-shaped pattern was found in air temperature, dew point and rainfall, but relative humidity showed a decreasing trend from May to October in the growing season. The highest air temperature, dew point and rainfall occurred in July, which presented great constraints on the photosynthetic physiology of poikilohydric terrestrial mosses [8]. Even though the highest air temperature in the present study was highly beyond the optimal temperature of previous research, these 12 moss species were more tuned to the mean air temperature as supported by previous research [30,31]. Multi-way ANOVAs revealed that seasonality, mainly the changes of rainfall and relative humidity pattern, had a significant impact on the photosynthetic performances of all 12 moss species. As bryophytes commonly develop the ability to lose most of the cell water without dying and resume normal functions during periods when external water is available, gaining positive carbon balance over wet–dry cycles, which is called desiccation tolerance, varied greatly between species [32,33]. Twelve moss species responded to the seasonal changes of cycles of drying and rehydrating and temperature in three different ways (Figure 12). 

*H. plumaeforme*, *P*. *crassicaulis* from 900 m and *P. inflexum* from 100 m were desiccation tolerant, and they well adapted to their habitat temperature and water patterns, which can be characterized by their water-conducting systems (endohydric: relying mostly on internal water transport and evolved vascular tissues; or mixohydric: employing both the ectohydric and endohydric water-conducting pathways in varying ratios) [32,33]. They gained the best photochemical quantum yield in the months of most rainfall or relative humidity and reached more than 75%, even 90% for *H. plumaeforme* and *P. inflexum* of the maximal photosynthesis rate even under the driest and lowest rainfall of October, despite a lower photosynthetic efficiency. The results of Y(NO) showed the reversed responses, having the non-regulated heat dissipation lowest and highest in the wet and dry months, respectively, and NPQ always had high and fast components of non-photochemical quenching values to dissipate the excess energy to protect the photosynthesis apparatus [20,21,34,35]. *H. plumaeforme* from 900 m showed high Stern-Volmer NPQ, which indicated the sufficient capacity of photoprotective reaction [36]. However, *P*. *crassicaulis* and *P. inflexum* exhibited a lower Stern-Volmer NPQ in contrast to *H. plumaeforme*. The different responses might reflect the differences of their ability to regulate the strong light [37]. Life forms of these three moss species might also contribute to their efficient acclimation to the changing abiotic environments. In the case of mat and weft, the capillary retention of water predominates with respect to physiological activity, especially photosynthesis, which enables species of these life forms, such as *P*. *crassicaulis* (mat) and *H. plumaeforme* (weft) in the present study, to extend their duration of being fully active beyond the time of rainfall [38]. In addition, *H. plumaeforme* had more than 50 mm thick of moss layers with the dead plant bodies underneath, which stored enough water for it to spend even during the drier days with less rainfall. The life form of *P. inflexum* is tall turf, growing in damp habitats, which had strong and fully-developed rhizoids on shoots deeply growing in the substrates [25]. For *P. inflexum* growing in warmer and drier lowlands, we did not find it at high elevations, which can be explained by habitat fragmentation and degradation [39]. 

The other nine species were sensitive to seasonality, especially to the months of lower rainfall and relative humidity when moss species were physiologically desiccated and inactive. They were ectohydric (relying mainly on water transport along the external surface of the plant by capillarity) or mixohydric to some extent and regulated their photochemical performances in two other ways. *T. glaucinoides*, *P. angustata* and *L. scabrum* were desiccation avoiders, who were well-tuned with seasonal changes of climatic factors, adjusting their photochemical efficiency accordingly. They obtained the highest Y(II) under the highest relative humidity and rainfall of July and lowest Y(II) (less 50% of values) under the driest and lowest rainfall of October, showing lower acclimation capability to dry conditions. This might reflect that water supply as opposed to temperature is a key factor affecting the physiological activity of moss species [40]. These three species had high Stern–Volmer type NPQ except in October, reaching a stationary phase with a statistically smaller and overall slower amplitude of the fast component, and instead, the quantum yield of those non-regulated were high, which strongly suggested the fraction of absorbed light energy neither drove photochemistry (Y(II)) nor thermally dissipated by rapid regulated Stern–Volmer type NPQ processes [35].

The other six species were susceptible to the seasonal changes of temperature and water patterns, which only reached less than 70% of maximal photochemical quantum yield of PS II even under the wettest and warmest environmental conditions. *B*. *buchananii* was especially sensitive to temperature and water pattern changes, which yielded only 33.6% of the maximal photosynthesis rate even under optimum months with the highest non-regulated losses of excitation energy Y(NO) and received the lowest and slowest component of the Stern–Volmer type NPQ. A high value of Y(NO) and low NPQ value also suggested insufficient capacity of photoprotective reactions, which would lead to photodamage when plants were exposed to high irradiance [40]. *A*. *julaceum*, *P*. *neesii*, *S*. *subhumile* and *P*. *pohliaecarpum* showed remarkably greater Y(NO) and lower Stern–Volmer NPQ than the other species. As we mentioned above, a much higher value of Y(NO) indicated that excess excitation energy reached the reaction centers, resulting in a strong reduction in PS II acceptors and photodamage [36,40], which partly contributed to the lower Y(II) values of the corresponding moss species. However, *L. boninense* presented higher non-regulated losses of excitation energy Y(NO) and higher and faster components of Stern–Volmer NPQ. *L. boninense* exhibited increased NPQ at an actinic irradiance of 1323 μmol/s/m^2^. This implied that *L. boninense* developed the ability to maintain photoprotection through regulated non-photochemical quenching [36,37].

## 4. Materials and Methods

### 4.1. Study Area

Tai Mo Shan is the highest peak in Hong Kong, 130 km south of the Tropic of Cancer (22°9′–22°33′ N, 113°50′–114°26′ E), with an elevation of 957 m, which features a humid subtropical climate with distinct hot humid and cool, dry seasons. It has an area of 1440 hectares and is situated in the Tai Mo Shan Country Park in the center of the New Territories, Hong Kong. Due to the height of the mountain, Tai Mo Shan is claimed to be Hong Kong’s most misty area, as it is often covered in clouds. It is not uncommon for temperatures to drop below the freezing point during the winter [41].

The vegetation types on our study sites along the elevation gradient change greatly from lowland to the peak. Dense broad-leaved lowland woodlands are mainly distributed in hilly areas below 400 m, with the dominant species *Glochidion hongkongense* and *Syzgium levinei* at 100 and 300 m, respectively. Dense broad-leaved low-hill forests are mostly located on uplands between 400 and 550 m. The species *Cinnamomum porrectum* is dominant at 500 m. Grasslands are widely found at upper slopes above 550 m to the peak. At 700 m, small montane shrub patches are distributed in the dense grasslands with the grass species *Miscanthus sinensis* being dominant. At 900 m and near the peak, montane shrub patches or montane forest patches exist, with *M. sinensis* again the commonest species. The area has become one of the major forest plantations with mainly native species selected in Hong Kong, starting from 2013 [42]. Trees planted here are mostly native species, such as *Ficus microcarpa*, *Endospermum chinense*, *Syzgium levinei*, *Antidesma bunius*, *Psychotria asiatica* and *Aquilaria sinensis*, with other non-natives, such as *Pinus massoniana*, *Acacia confusa*, *Lophostemon confertus* and *Melaleuca quinquenervia*. Forests are limited to a maximum altitude of 550 m, while the upper slopes are dominated by shrubs and grasses [43].

### 4.2. Study Species

**Figure 13:**
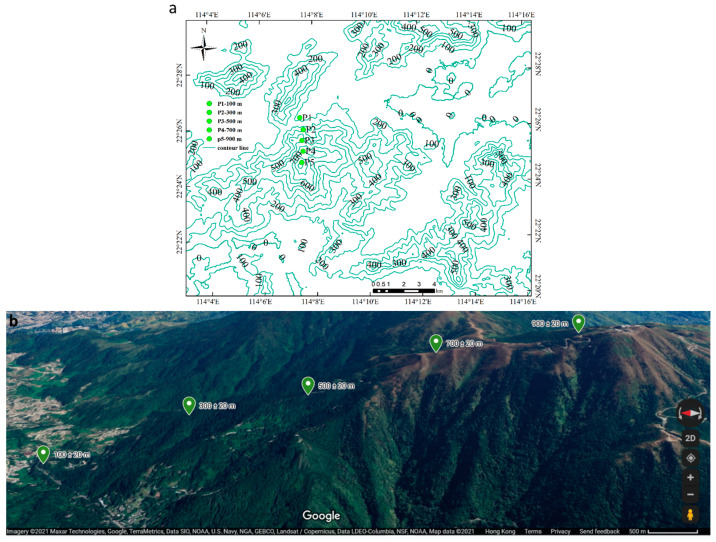
Map showing the study site on Tai Mo Shan in the central part of Hong Kong (**a**), Google map showing the five sampling points on Tai Mo Shan from 100 to 900 m with elevation intervals of 200 m (**b**).

Field studies were carried out during the growing season (from May to October) of 2019. Five elevations were selected from the northern aspect of Tai Mo Shan, with altitudinal intervals of 200 m from 100 ± 20 m to 900 ± 20 m. Twelve most common and dominant moss species at the five study elevations were selected as study materials (Table 1). Figure 13 shows the 5 study sites.

### 4.3. Chlorophyll Fluorescence Measurements

At the end of each month, rapid light curve (RLC) was measured using a Photosynthesis Yield Analyzer MINI-PAM-II in situ (MINI-PAM II Series Chlorophyll Fluorescence System, Heinz-Walz Instruments, Effeltrich, Germany) according to the manufacturer’s instructions. The light curve program exposed the sample to stepwise increasing intensities of actinic illumination [44]. In RLC, the time interval of each light step was short (down to 10 s), even though full equilibration of photosynthetic reactions was not reached within an illumination interval. However, the time interval of RLC is short enough so that RLC data provide information on the present acclimation state of photosynthesis in the natural environment. After field measurements, we collected and transferred the 12 moss species samples to the laboratory for induction curve and recovery measurements for control and dark-acclimatized for at least half an hour, which were remoistened if necessary. For induction curve measurements, a dark leaf clip was connected with a Leaf-Clip Holder 2030-B. Parameters were automatically calculated by the independently operated MINI-PAM-II or by software WinControl-3. They included complementary PS II yields, Fv/Fm (maximal photochemical yield of PS II), Y(II) (effective photochemical yield of PS II), Y(NPQ) (regulated energy losses of excitation energy by heat dissipation involving ΔpH- and zeaxanthin-dependent mechanisms), Y(NO) (non-regulated losses of excitation energy including heat dissipation and fluorescence emission) and Stern–Volmer type NPQ (parameters of non-photochemical quenching).

Complementary PS II quantum yields were used to analyze the partitioning of absorbed light energy in photosynthetic organisms [45]. For instance, a much higher value of Y(NO) than Y(NPQ) denotes that excess excitation energy reached the reaction centers, resulting in a strong reduction in PS II acceptors and photodamage, e.g., via formation of reactive oxygen species [36,40]. In contrast, high Y(NPQ) indicates that excess excitation energy is dissipated via regulated mechanisms at the antenna level and that photosynthetic energy fluxes are well-regulated. The most frequently used non-photochemical quenching parameter, the Stern–Volmer type quenching coefficient, NPQ, is sensitive to both regulated and constitutive thermal energy dissipation, reflecting the magnitude of the regulated component of non-photochemical quenching [46,47,48].

(1) $$\frac{\mathrm{Fv}}{\mathrm{Fm}}=\frac{\left(\mathrm{Fm}-F0\right)}{\mathrm{Fm}}$$

(2) $$Y\left(\mathrm{II}\right)=\frac{\mathrm{Fm}\prime -F}{\mathrm{Fm}\prime }$$

(3) $$Y\left(\mathrm{NPQ}\right)=\frac{F}{\mathrm{Fm}\prime }-\frac{F}{\mathrm{Fm}}$$

(4) $$Y\left(\mathrm{NO}\right)=\frac{F}{\mathrm{Fm}}$$

(5) $$\mathrm{NPQ}=\frac{\mathrm{Fm}-\mathrm{Fm}\prime }{\mathrm{Fm}\prime }$$

(6) 1=Y(II)+Y(NO)+ Y(NPQ)

### 4.4. Environmental and Climatic Factors

Environmental and climate data (including air temperature, relative humidity, rainfall, dew point) at each site were obtained from the local weather station if possible. For two elevations (500 and 700 m) from the northern aspect, of which weather data were not available, climate parameters were measured at the self-deployed weather stations. Three mini weather stations were set up at each elevation to collect air temperature, dew point, relative humidity and rainfall.

### 4.5. Statistical Analysis

All environmental variables were averaged for each elevation. All statistical tests were performed using SPSS (version 26.0; IBM, Armonk, NY, USA). Data were checked for deviations from normality and homogeneity of variance before statistical analysis. ANOVAs with Tukey’s post hoc tests were performed to assess significant differences among different observation times. Differences in the Y(II), Y(NO), NPQ and environmental factors among different elevations and sampling months were analyzed using multi-way ANOVAs.

## 5. Conclusions

These 12 moss species developed different strategies actively or passively to adapt to the challenges of the cycle of drying and rehydrating and hot environments. We found that only *H. plumaeforme*, *P*. *crassicaulis* and *P. inflexum* were well acclimatized to their habitat, even though they still experienced a lower photochemical efficiency. Their life forms were also optimized to adapt to the habitats. However, the other nine species were mostly sensitive to seasonality, and *A*. *julaceum*, *P*. *neesii*, *S*. *subhumile*, *P*. *pohliaecarpum* and *L*. *boninense*, especially *B*. *buchananii* were ideal as bioindicators for climate change. Even though they responded differently, all these moss species developed suitable regulated and non-regulated strategies to avoid the detrimental effect of abiotic stresses. However, all species have limitations to their adaptive ability of response to changing environments, and the limits are unlikely to improve for species already experiencing the abiotic stresses close to their tolerance ceiling. As tropics are expected to become hotter and drier, many moss species might be negatively affected or even at the risk of extinction, which makes tropical regions hotspots to explore the potential influences of global climate change on the non-vascular plants and even other biomes.

## Figures and Tables

**Table 1:** Study moss species at 5 study elevations in the experiment (listed in descending order of their relative field abundance).

Altitude (m)	Species	Family	Life Form
900	*Hypnum plumaeforme*	Hypnaceae	Weft
	*Brachythecium buchananii*	Brachytheciaceae	Mat
	*Thuidium glaucinoides*	Thuidiaceae	Weft
	*Anomobryum julaceum*	Bryaceae	Cushion
	*Pterobryopsis crassicaulis*	Pterobryaceae	Mat
	*Pseudosymblepharis angustata*	Pottiaceae	Turf
700	*Pogonatum neesii*	Polytrichaceae	Turf
	*Leucobryum scabrum*	Leucobryaceae	Cushion
500	*Sematophyllum subhumile*	Sematophyllaceae	Mat
	*Leucobryum boninense*	Leucobryaceae	Cushion
300	*Pseudotaxiphyllum pohliaecarpum*	Hypnaceae	Mat
100	*Pogonatum inflexum*	Polytrichaceae	Turf

## Data Availability

The authors confirm that the data supporting the findings of this study are available within the article and its appendices.

## Appendix A

**Table A1:** Multi-way ANOVAs for environmental factors at 5 elevations during observing months.

Altitude (m)	Measure	df	F	*p*
900	Air temperature	5	252.944	<0.001
	Dew point	5	460.868	<0.001
	Relative humidity	5	122.333	<0.001
	Rainfall	5	5919.829	<0.001
700	Air temperature	5	158.187	<0.001
	Dew point	5	147.530	<0.001
	Relative humidity	5	254.240	<0.001
	Rainfall	5	3020.540	<0.001
500	Air temperature	5	150.962	<0.001
	Dew point	5	310.839	<0.001
	Relative humidity	5	71.350	<0.001
	Rainfall	5	3661.869	<0.001
300	Air temperature	5	219.498	<0.001
	Dew point	5	670.124	<0.001
	Relative humidity	5	75.954	<0.001
	Rainfall	5	6.106	<0.01
100	Air temperature	5	220.179	<0.001
	Dew point	5	930.688	<0.001
	Relative humidity	5	100.763	<0.001
	Rainfall	5	4624.528	<0.001

## Appendix B

**Table A2:** Multi-way ANOVAs for Y(II) of 12 selected moss species from 5 elevations during observing months.

Altitude (m)	Species Name	df	F	*p*
900	*Hypnum plumaeforme*	6	54.765	<0.001
	*Brachythecium buchananii*	6	4476.718	<0.001
	*Thuidium glaucinoides*	6	1585.716	<0.001
	*Anomobryum julaceum*	6	1345.491	<0.001
	*Pterobryopsis crassicaulis*	6	312.981	<0.001
	*Pseudosymblepharis angustata*	6	6982.168	<0.001
700	*Pogonatum neesii*	6	1035.626	<0.001
	*Leucobryum scabrum*	6	2610.740	<0.001
500	*Sematophyllum subhumile*	6	2165.445	<0.001
	*Leucobryum boninense*	6	3889.871	<0.001
300	*Pseudotaxiphyllum pohliaecarpum*	6	1463.714	<0.001
100	*Pogonatum inflexum*	6	23.889	<0.001

## Appendix C

**Table A3:** Multi-way ANOVAs for Y(NO) and NPQ of 12 selected moss species from 5 elevations during observing months.

Altitude (m)	Species Name	df	F	*p*
900	*Hypnum plumaeforme*			
	Y(NO)	6	54.765	<0.001
	NPQ	5	683.014	<0.001
	*Brachythecium buchananii*			
	Y(NO)	6	4476.718	<0.001
	NPQ	5	101.123	<0.001
	*Thuidium glaucinoides*			
	Y(NO)	6	1585.716	<0.001
	NPQ	5	22,168.897	<0.001
	*Anomobryum julaceum*			
	Y(NO)	6	1345.491	<0.001
	NPQ	5	8337.127	<0.001
	*Pterobryopsis crassicaulis*			
	Y(NO)	6	299.129	<0.001
	NPQ	5	370.082	<0.001
	*Pseudosymblepharis angustata*			
	Y(NO)	6	6982.168	<0.001
	NPQ	5	47,520.709	<0.001
700	*Pogonatum neesii*			
	Y(NO)	6	1035.626	<0.001
	NPQ	5	2354.901	<0.001
	*Leucobryum scabrum*			
	Y(NO)	6	2610.740	<0.001
	NPQ	5	1870.289	<0.001
500	*Sematophyllum subhumile*			
	Y(NO)	6	2165.445	<0.001
	NPQ	5	2333.928	<0.001
	*Leucobryum boninense*			
	Y(NO)	6	4288.060	<0.001
	NPQ	5	15,013.965	<0.001
300	*Pseudotaxiphyllum pohliaecarpum*			
	Y(NO)	6	1463.714	<0.001
	NPQ	5	212.636	<0.001
100	*Pogonatum inflexum*			
	Y(NO)	6	23.889	<0.001
	NPQ	5	446.211	<0.001
